# Increased PM_2.5_ Caused by Enhanced Fireworks Burning and Secondary Aerosols in a Forested City of North China During the 2023–2025 Spring Festivals

**DOI:** 10.3390/toxics13121009

**Published:** 2025-11-21

**Authors:** Qingxia Ma, Guoqing Zhao, Kaixin Cheng, Yunfei Wu, Renjian Zhang, Lei Gu, Jing Xue, Wanfu Feng, Jiliang Zhou, Xinzhi Shen, Dexin Liu

**Affiliations:** 1 College of Geographical Science, Faculty of Geographical Science and Engineering, Henan University, Zhengzhou 450046, China; mqx@henu.edu.cn (Q.M.); zgq104753230269@henu.edu.cn (G.Z.); 7080@henu.edu.cn (K.C.); hedagulei1981@vip.henu.edu.cn (L.G.); 2 Key Laboratory of Geospatial Technology for the Middle and Lower Yellow River Regions, Ministry of Education, Henan University, Kaifeng 475004, China; 3 Henan Key Laboratory of Air Pollution Control and Ecological Security, Kaifeng 475004, China; 4 State Key Laboratory of Atmospheric Environment and Extreme Meteorology (AEEM), Institute of Atmospheric Physics, Chinese Academy of Sciences, Beijing 100029, China; wuyf@mail.iap.ac.cn; 5 Institute of Atmospheric Physics, Chinese Academy of Sciences, Beijing 100029, China; zrj@tea.ac.cn; 6 School of Ecology and Environment, Northwestern Polytechnical University, Xi’an 710129, China; xuejing0089@nwpu.edu.cn; 7 The Forest Science Research Institute of Xinyang, Xinyang 464031, China; lksfwf@163.com; 8 Henan Jigongshan Forest Ecosystem Observation and Research Station, Xinyang 464031, China; 9 Xinyang Ecological Environment Monitoring Center of Henan Province, Xinyang 464000, China; zhoujiliang888@163.com (J.Z.); sxz6719@163.com (X.S.)

**Keywords:** PM_2.5_, source apportionment, secondary aerosol formation, fireworks emission

## Abstract

Fireworks burning (FB) constitutes a major but short-lived source of PM_2.5_ during the Chinese Spring Festival, significantly deteriorating air quality in certain regions. This study was conducted to evaluate its impact through real-time monitoring of PM_2.5_ chemical compositions in a forestry city (Xinyang) during the pre-fireworks and fireworks periods at the Spring Festival of 2023–2025. During the fireworks period, PM_2.5_ concentrations increased by 10.5–226.4% compared to pre-fireworks levels, of which the concentrations of secondary inorganic aerosols (SIA), K and Cl^−^ rose by 1.6–4.8, 1.9–14.7 and 1.5–8.1 times, and they accounted for 33.2–47.7%, 6.7–12.5% and 3.8–6.4% of PM_2.5_, respectively. Correspondingly, PM_2.5_/CO and SIA/CO ratios in 2023–2025 elevated by factors of 1.4–2.3 and 1.1–3.4, indicating distinct enhancements in secondary inorganic aerosols formation. Additionally, acidity of PM_2.5_, RH and O_x_ also increased during fireworks. Collectively, higher sulfur and nitrogen oxidation ratios (SOR and NOR) during the fireworks period under the combined effects of high RH, O_x_ and acidity conditions indicated a greater conversion of secondary inorganic aerosols. Positive Matrix Factorization (PMF) analysis confirmed that FB and secondary aerosols (SA) source levels during fireworks increased by 2.5–19.3 and 1.9–4.4 times compared to pre-fireworks values. This study underscores the need for implementing stringent management of fireworks and secondary formation mitigation to reduce PM_2.5_ concentrations during the Spring Festival.

## 1. Introduction

Fireworks used in global celebrations adversely affect air quality, consequently imposing a substantial burden on governments [1]. This is especially evident in China during the Spring Festival, where nationwide displays significantly elevate PM_2.5_ levels and substantially deteriorate air quality [2]. For instance, PM_2.5_ concentrations on firework days (248.9 μg m^−3^) was greatly higher than before firework days (90.8 μg m^−3^) in Beijing during the Spring Festival, with firework emissions identified as the primary contributor [3]. A similar pattern was observed in the United States, where fireworks combustion raised daily PM_2.5_ levels by 42% during the Independence Day [4]. Although fireworks burning activities are relatively short-lived in duration, the frequent use of fireworks during festivals poses irreversible risks to humans, by inhalation of high concentration particulates containing harmful chemicals (including heavy metals and polycyclic aromatic hydrocarbons) [5].

Numerous studies have demonstrated confirmed that firework combustion releases substantial gaseous pollutants (including SO_2_ and NO_2_) and particulate matter (including water-soluble ions, metallic elements, and organic compounds) [6,7,8]. Cao et al. (2018) found that the concentrations of SO_2_ and CO during the Spring Festival were 9.2 times and 5 times higher than the background levels, respectively [9]. Concurrently, Hu et al. (2019) found the highest values of K^+^, Cl^−^ and SIA during the fireworks period in Beijing [10]. In addition, the combustion of fireworks and firecrackers also affects the aerosols’ acidity and alters the gas-particle partitioning of semi-volatile substances, thereby influencing the formation of secondary inorganic aerosols [11,12]. Wu et al. (2018) found that fine nitrate was mainly formed by heterogeneous reaction of HNO_3_ and NH_3_, while sulfate was mainly formed by adsorption on wet particles (such as KCl) produced by SO_2_ during firework activities [13]. Tian et al. (2014) quantified 29.7% contribution of fireworks to PM_2.5_ during the heavy-fireworks period by PMF model [14].

Since 2015, numerous cities in China have implemented regulations to restrict the discharge of fireworks during the Spring Festival, aiming to alleviate severe PM_2.5_ pollution [15]. Despite the marked improvements in air quality resulting from stringent clean-air measures, certain regions have recently loosened these restrictions, which posed critical challenges for air quality improvement strategy [16]. Xinyang, situated in the southern part of the North China Plain (NCP), is a representative forested city with a forest coverage rate of 36.2% [17]. Nevertheless, the prevalent phenomena of setting off fireworks in Xinyang, combined with its basin topography and stagnant winter meteorological conditions, still exacerbated the accumulation of PM_2.5_ [18]. In response, Xinyang launched an air pollution prevention and control initiative on fireworks and firecrackers in 2018 (https://www.xinyang.gov.cn/2018/04-02/554574.html, accessed on 3 October 2025). However, few studies have assessed the specific impact of firework emissions on air quality during the Spring Festival in Xinyang.

The study aims to address four major objectives: (1) quantify the temporal variations in air pollutants across the pre-fireworks, fireworks and post-fireworks phases; (2) investigate the impacts of fireworks on PM_2.5_ chemical composition and aerosol acidity; (3) analyze the formation of NO_3_^−^ and SO_4_^2−^ with intense firework discharge; and (4) estimate the source contribution and profile of the selected chemical compounds by the Positive Matrix Factorization (PMF) model. This approach enables a clear identification of the drivers of PM_2.5_ increases and compositional changes during firework events, thereby providing essential scientific support for strengthening pollution control in other regions severely affected by fireworks.

## 2. Materials and Methods

### 2.1. Observational Site

**Figure 1:**
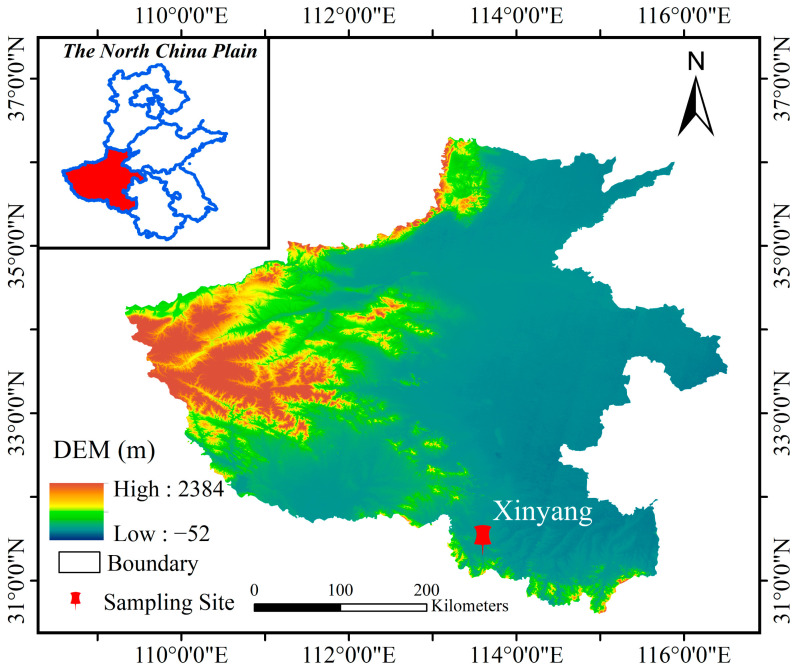
Map of the sampling site, was created using ArcGIS (ArcMap) v10.7. The geospatial data used were sourced from the National Platform for Common Geospatial Information Services (https://www.tianditu.gov.cn/, accessed on 3 October 2025).

Integrated measurements were carried out in a forestry city, Xinyang (called XY; 114°09′ E, 32°15′ N) of Henan Province, situated in southern NCP (Figure 1). The study period spanned from 20 to 28 January 2023, 16 to 20 February 2024 and 27 January to 4 February 2025, respectively. The location is surrounded by residential areas and lacks industrial emissions. Given the extensive firework displays that occurred in the Spring Festival to celebrate the Lunar New Year, the monitoring period for each year was strategically categorized into three phases: pre-fireworks phase (from the day before New Year’s Eve to 10:00 on New Year’s Eve), fireworks phase (from 11:00 on New Year’s Eve to the third day of the lunar new year) and post-fireworks phase (from the fourth to the seventh day), respectively.

### 2.2. Sample Collection and Laboratory Analysis

The monitoring station was established on the roof of the Xinyang Museum, approximately 18 m above ground level. Between 2023 and 2025, hourly PM_2.5_ concentrations were monitored with an ambient particulate matter analyzer (LGH-01E, Anhui Landun Photoelectron Co., Ltd., Lu’an, Anhui, China), with an airflow rate of 16.7 L/min. The product used the β-ray method, combined with a dynamic moderate control system and dynamic digital filtering technology, to achieve continuous monitoring of PM_2.5_. Calibration followed the HJ 817-2018 standard [19]. Concurrently, gaseous pollutants including SO_2_, NO_2_ and O_3_ were measured simultaneously with online analyzers (LGH-210, LGH-220 and LGH-240, Anhui Landun Photoelectron Co., Ltd., Lu’an, Anhui, China), with a time resolution of 1 h. These instruments enabled continuous monitoring based on ultraviolet fluorescence, chemiluminescence and ultraviolet absorption methods, respectively, and were calibrated according to the GB/T 36090-2018 standard [20]. The inorganic water-soluble ions (NH_4_^+^, NO_3_^−^, SO_4_^2−^, K^+^, Ca^2+^, Mg^2+^, Na^+^ and Cl^−^) were analyzed by ion chromatography (URG-9000, Thermo Fisher Scientific, Waltham, MA, USA), with a flow rate of 3 L/min and a time resolution of 1 h. Additionally, the sum of the mass concentrations of SO_4_^2−^, NO_3_^−^ and NH_4_^+^ was defined as secondary inorganic aerosols (SIA) in this study. Detailed instrument principles and quality control procedures were adopted from prior research [21]. Elemental components in PM_2.5_ (including K, Ca, Fe, Pb, Zn, Mn, Se, Hg, Cr, Cr, Cd, Cu, Ni, Ti, Sb, Sn, V, Ba, As, Co, Mo, Ag, Sc, Tl, Pd, Br, Te, Ga, Cs, Si and Al) were quantified with an online metal monitor (AMMS-100, Focused Photonics Inc., Hangzhou, Zhejiang, China), with a flow rate of 16.7 L/min and a time resolution of 1 h. Particles were collected on a 2 µm Teflon filter tape and the calibration of the elements was conducted using the NIST SRM 2783 reference material. Meteorological parameters (wind speed, wind direction, relative humidity, temperature) were recorded every minute by a compact weather station (LGH-01C, Anhui Landun Photoelectron Co., Ltd., Lu’an, Anhui, China, and hourly averages of meteorological conditions were applied, with the calibration based on the QX/T 291-2015 standard [22].

To ensure data quality and accuracy, all instruments underwent weekly calibration using relevant reference standards. Furthermore, a comprehensive quality assurance/quality control (QA/QC) protocol was implemented throughout the monitoring period from 2023 to 2025, which included the concurrent collection of field blanks and parallel samples.

### 2.3. Analysis Methods

#### 2.3.1. Acidity of PM_2.5_ Acidity

The ion balance is an effective approach to assess the acidity and alkalinity of atmospheric aerosols [23]. The anion equivalence (AE) and cation equivalence (CE) are estimated using Equations (1) and (2):

(1) $$\mathrm{AE}\;=\;\frac{\left[F^{-}\right]}{19}+\frac{\left[{\mathrm{Cl}}^{-}\;\right]}{35.5}+\frac{\left[{\mathrm{SO}}_{4}^{2-}\;\right]}{48}+\frac{\left[{\mathrm{NO}}_{3}^{-}\right]}{62}\;$$

(2) $$\mathrm{CE}=\frac{\left[{\mathrm{Na}}^{+}\right]}{23}+\frac{\left[{\mathrm{NH}}_{4}^{+}\;\right]}{18}+\frac{\left[\;K^{+}\right]}{39}+\frac{\left[{\mathrm{Mg}}^{2+}\right]}{12}+\frac{\left[{\mathrm{Ca}}^{2+}\right]}{20}$$

where [X] represents the observed concentration of each element. The AE/CE ratio is commonly used to evaluate aerosol acidity: the ratio less than 1 indicates alkaline conditions, equal to 1 indicates neutrality, and greater than 1 indicates acidic conditions [24].

#### 2.3.2. Nitrogen and Sulfur Oxidation Ratios

The sulfur and nitrogen oxidation ratios (SOR and NOR) are key indicators to evaluate the formation of secondary inorganic aerosols [25]. The NOR and SOR are calculated using Equations (3) and (4).

(3) $$\mathrm{NOR}=\frac{\left[{\mathrm{NO}}_{3}^{-}\right]}{\left[{\mathrm{NO}}_{2}\right]+[{\mathrm{NO}}_{3}^{-}]}\;$$

(4) $$\mathrm{SOR}=\frac{\left[{\mathrm{SO}}_{4}^{2-}\right]}{\left[{\mathrm{SO}}_{2}\right]+[{\mathrm{SO}}_{4}^{2-}]}\;$$

where the concentrations of SO_2_, NO_2_, NO_3_^−^ and SO_4_^2−^ are expressed as molar concentrations.

#### 2.3.3. Source Apportionment of PM_2.5_ Positive Matrix Factorization (PMF) Model

The Positive Matrix Factorization (PMF) model (US EPA PMF 5.0) is an analytical tool, which is widely used to identify PM_2.5_ pollution sources and quantify their contributions based on the compositional profiles [26,27]. In this study, PMF method was applied to analyze the source apportionment of PM_2.5_, while multiple PMF runs were performed based on Q-values, error estimation, and source chemical fingerprints to determine the optimal factor number [28]. Moreover, the reliability of PMF model was also confirmed by the strong correlation (Figure S2) between observed and reconstructed PM_2.5_ (the sums of NH_4_^+^, NO_3_^−^, SO_4_^2−^, Ca^2+^, K^+^, Mg^2+^, Cl^−^, OC, EC, Fe, Pb, Zn, Mn, Se, Cr, Cu, Ti, Ba, As, Br, Cs and Si) over three years. More details are depicted in the Supplementary Materials.

## 3. Results and Discussion

### 3.1. The Variations in Meteorological Conditions and Air Pollutants During the Spring Festival

**Figure 2:**
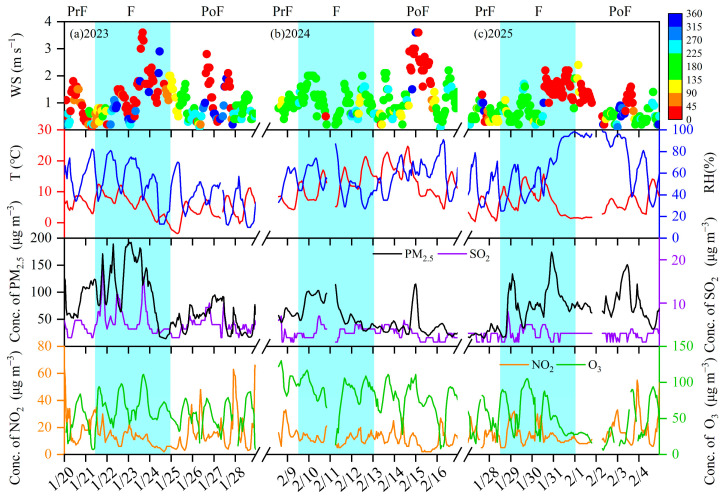
Time series of PM_2.5_, air pollutants and meteorological conditions during pre-fireworks, fireworks and post-fireworks periods in XY of 2023–2025: (**a**) 2023; (**b**) 2024; (**c**) 2025. The PrF, F and PoF are the abbreviations of pre-fireworks, fireworks and post-fireworks periods, respectively. The Ws represents the wind speeds (Ws), and the color scale stands for the wind direction.

Figure 2a–c and Tables S1–S3 illustrate the variations of PM_2.5_, precursor concentrations and meteorological conditions in XY during the pre-fireworks, fireworks and post-fireworks periods. The PM_2.5_ concentrations were 113.6, 66.2 and 81.2 μg m^−3^ during the fireworks period from 2023 to 2025, which were 1.3, 1.1 and 3.3, and 2.3, 1.8 and 1.1 times higher than those at the pre-fireworks and post-fireworks periods, respectively. Previous research conducted in Tianjin, Beijing and Zhengzhou also revealed increased PM_2.5_ mass concentrations caused by fireworks events [29,30,31].

The concentration of SO_2_ initially increased from 4.0 (2023), 2.7 (2024) and 2.3 (2025) μg m^−3^ pre-fireworks to 6.2, 3.5 and 3.0 μg m^−3^ during fireworks, and subsequently dropped by 22.9%, 18.5% and 11.6% post-fireworks. This was consistent with prior research, which found that the extensive fireworks burning resulted in sharply elevated concentrations of SO_2_ during the Spring Festival at Shanghai [32]. The NO_2_ levels decreased by 75.0% during fireworks in 2023, compared with the pre-fireworks period concentration of 21.7 μg m^−3^, then greatly increasing by 41.6% post-fireworks. In 2024, its concentration declined from 19.1 μg m^−3^ pre-fireworks to 12.1 μg m^−3^ during fireworks and further to 10.9 μg m^−3^ post-fireworks. The generally lower NO_2_ concentrations during fireworks were closely associated with reduced vehicle usage for celebrating the Spring Festival [33]. This result was consistent with prior research, which evaluated the air quality among 366 sites in China and found the reductions of NO_2_ were positively linked with the decreased emissions from vehicles [34]. In 2025, the variations in NO_2_ values were stable at pre-fireworks (12.4 μg m^−3^) and fireworks periods (12.1 μg m^−3^), while they increased to 16.5 μg m^−3^ post-fireworks. In terms of O_3,_ its concentration reached 68.0 μg m^−3^ in 2023 and 58.0 μg m^−3^ in 2025 during fireworks, 1.3–1.7 and 1.4–1.6 times higher than those during fireworks and post-fireworks periods, respectively, suggesting the enhanced oxidating capacity in ambient air was affected by fireworks [35]. However, the O_3_ value in 2024 decreased from 103.2 μg m^−3^ pre-fireworks to 88.4 μg m^−3^ during fireworks, and subsequently to 74.8 μg m^−3^ post-fireworks.

Meteorological conditions exhibited notable variations across three periods. Although the average wind speeds (Ws) consistently increased during the fireworks period in three years, it remained below 2 m s^−1^, reflecting calm wind conditions. Simultaneously, the average relative humidity (RH) maintained at high levels, ranging from 51.6% to 60.9% during the fireworks in three years. A consistent rise in average temperature (T) was also observed during the fireworks events in three years. Specifically, from 2023 to 2025, the average T enhanced from 3.8 to 6.1 °C pre-fireworks to 6.5–12.4 °C during fireworks from 2023 to 2025, indicating relatively warm weather conditions.

### 3.2. Impacts of Fireworks on Chemical Components and Aerosol Acid During the Spring Festival

#### 3.2.1. Changes in Elemental Compositions and SIA

**Figure 3:**
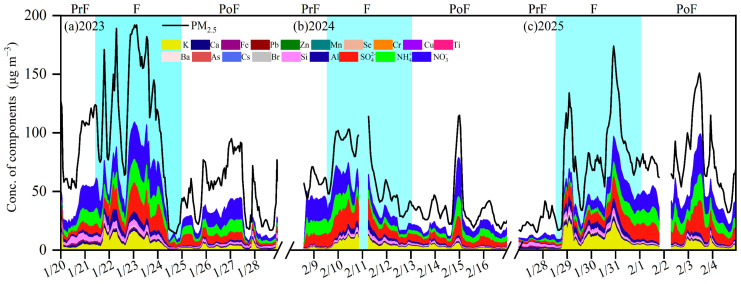
Time series of PM_2.5_ and chemical compositions during pre-fireworks, fireworks and post-fireworks periods in XY over three years: (**a**) 2023; (**b**) 2024; (**c**) 2025. The PrF, F and PoF are the abbreviations of pre-fireworks, fireworks and post-fireworks periods, respectively.

Figure 3 showed the time series of PM_2.5_ and its chemical components, including sulfate (SO_4_^2−^), nitrate (NO_3_^−^), ammonium (NH_4_^+^), potassium (K), calcium (Ca), iron (Fe), lead (Pb), zinc (Zn), manganese (Mn), selenium (Se), chromium (Cr), copper (Cu), titanium (Ti), barium (Ba), arsenic (As), cesium (Cs), bromine (Br), silicon (Si), and aluminum (Al). Intensive fireworks burning activities substantially elevated PM_2.5_ concentrations and altered its chemical compositions, such as secondary inorganic aerosols (SIA), K and Cl^−^ [36,37,38]. Common oxidizers used in fireworks included K compounds (such as KNO_3_, KClO_3_, KClO_4_, K_2_CrO_4_ and K_2_Cr_2_O_7_) [39]. Cl^−^ serves as a key tracer for firework emissions, primarily employed in generating diverse color effects during combustion [40]. As shown in Figure 3 and Tables S1–S3, the concentrations of Cl^−^ and K were 0.6–4.2 and 0.7–4.0 μg m^−3^ pre-fireworks, accounting for 2.4–4.8% and 2.3–4.5% to PM_2.5_, respectively. Their contributions increased to 3.8–6.4% and 6.7–12.5% during fireworks, while they dropped to 2.6–5.3% and 4.2–5.8% post-fireworks, underscoring the predominant influence of fireworks on their atmospheric abundance. A previous study conducted in Linyi similarly found increases in Cl^−^ and K by factors of 3.6 and 11.0 during the fireworks burning period compared to non-fireworks burning period [41]. Moreover, compared with experiments from Changzhou and Zhengzhou, Cl^−^ and K values in this study were considerably higher, while K concentrations were markedly lower than those in Zaozhuang, Shandong Province [42,43,44]. This discrepancy might be attributed to Zaozhuang’s abundant mineral resources of coal, which released significant quantities of K into the ambient environment through mining and smelting processes [45].

The concentration of SIA (44.3 μg m^−3^) at fireworks period in 2023 was 1.6 and 2.3 times higher than the pre-fireworks and post-fireworks values. In 2024, the value of SIA was 39.3 μg m^−3^ pre-fireworks, decreasing by 19.7% during fireworks, and further by 42.6% post-fireworks. The decreased SIA level during the fireworks phases was extremely affected by the reduced RH, increased T and stronger Ws (Table S5, *p* < 0.01). In 2025, the contribution of SIA (5.6 μg m^−3^) to PM_2.5_ reached 22.6% pre-fireworks, increasing to 33.2% during fireworks, subsequently surged to 52.1% post-fireworks, suggesting markedly enhanced secondary formation under intensive fireworks emissions. Generally, the sum of contribution from SIA, Cl^−^ and K to PM_2.5_ during the fireworks period accounted for 51.7% to 60.0% over three years, demonstrating the important role of secondary inorganic aerosols and firework-related species in increased PM_2.5_.

**Figure 4:**
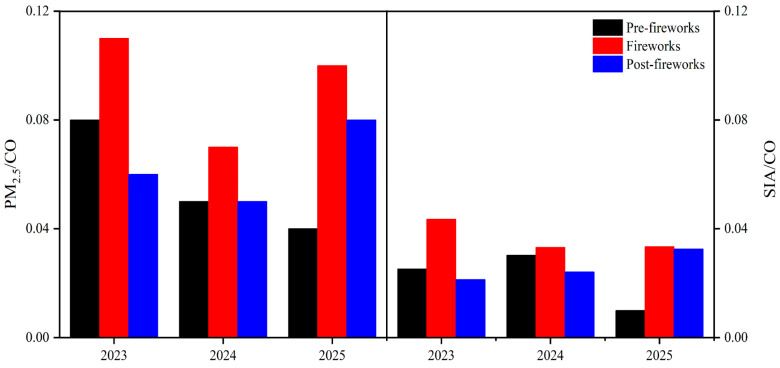
The PM_2.5_/CO and SIA/CO ratios during pre-fireworks, fireworks and post-fireworks periods in XY of 2023–2025.

Notably, the massive fireworks discharge led to significant increases in both the concentrations of PM_2.5_ and SIA, while the contribution of SIA to PM_2.5_ increased gradually (Figure S4). Similarly, Feng et al. (2016) analyzed the variations in SIA during the Spring Festival in Xinxiang and found a 36.5% increase in NO_3_^−^ after intensive combustion of fireworks [46]. CO was used as a tracer for primary particulate matter [47]. The PM_2.5_/CO ratio was frequently used to exclude the influence of meteorological conditions, reflecting the contributions from emissions and chemical transformations [48,49]. The rising PM_2.5_/CO ratios represented a better contribution of secondary formation to PM_2.5_ [50,51]. The robust correlation was found between PM_2.5_ and CO (Table S6, R^2^ = 0.67–0.84 in 2023–2025, *p* < 0.01). During the fireworks periods of 2023–2025, the highest PM_2.5_/CO ratios were 1.1 × 10^−1^, 7.0 × 10^−2^ and 1.0 × 10^−1^ (Figure 4), exceeding the levels both in pre-fireworks and post-fireworks phases by factors of 1.4–2.3 and 1.4–2.0, respectively. The results suggested a greater contribution of secondary formation to PM_2.5_. Moreover, the SIA/CO ratio was employed to quantify the contribution of SIA transformation. From 2023 to 2025, SIA/CO ratios were about 3.3 × 10^−2^–4.4 × 10^−2^ at the fireworks periods (Figure 4), and were 1.1–3.4 and 1.0–2.0 times higher than those in the pre-fireworks and post-fireworks phases, which suggested a better formation of secondary inorganic aerosols, supported by their strong correlations (Table S7, R^2^ = 0.31–0.72 in 2023–2025, *p* < 0.01). In addition, large quantities of precursor pollutants (including SO_2_ and NO_2_) generated by fireworks [52] underwent homogeneous and heterogeneous processes, further affecting SIA/CO ratios [53,54]. These reactions markedly amplified the contribution of fireworks to secondary aerosol formation. Moreover, stagnant meteorological conditions during the Spring Festival in XY also exerted pronounced influences on homogeneous and heterogeneous reactions.

However, the increasing pattern between PM_2.5_/CO and SIA/CO ratios was observed during the fireworks period across three years. While the difference between these ratios pre-fireworks and during fireworks was the lowest in 2024, these ratios in 2024 were also lower than those in 2023 and 2025. The Xinyang government adopted control measures on 24 December 2023 (https://www.xyxww.com.cn/jhtml/xinyang/353215.html, accessed on 7 November 2025) and 31 January 2024 (https://sthjj.xinyang.gov.cn/2024/01-31/130288.html, accessed on 7 November 2025) to address heavy pollution during the Spring Festival. Concurrently, a notable temperature rise (from 5.9 °C to 12.4 °C) occurred during the fireworks period in 2024, which might enhance vertical convection, thereby facilitating the dispersion of PM_2.5_ and its components [55]. Under the synergistic effects of these factors, the differences of PM_2.5_/CO and SIA/CO between the pre-fireworks and fireworks periods in 2024 were reduced, and led to correspondingly lower PM_2.5_/CO and SIA/CO ratios compared to 2023 and 2025. In summary, the increased PM_2.5_/CO and SIA/CO ratios from 2023 to 2025 underscored the necessity of implementing targeted controls on secondary emissions for effectively curbing the growth of PM_2.5_.

#### 3.2.2. Acidity of PM_2.5_

**Figure 5:**
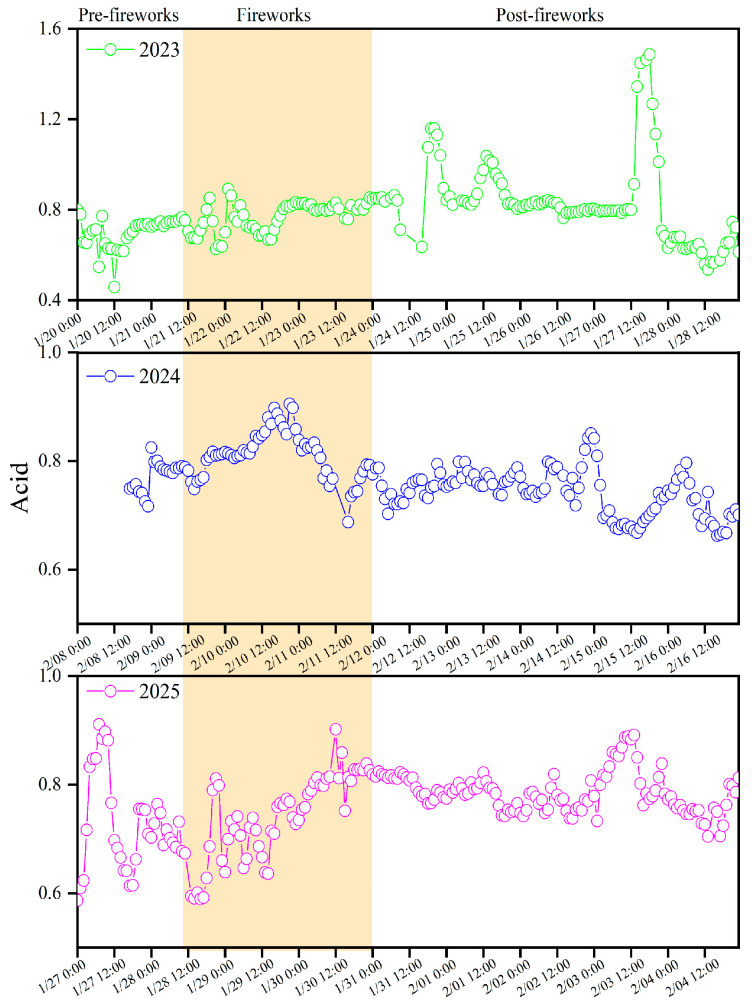
Variations in acid during pre-fireworks, fireworks and post-fireworks periods in XY from 2023 to 2025.

The cation–anion balance was an effective approach for assessing the acidity and alkalinity of atmospheric aerosols [56]. Anions such as SO_4_^2−^, NO_3_^−^, F^−^ and Cl^−^ contributed to aerosol acidity, while cations NH_4_^+^, K^+^, Na^+^ and Ca^2+^ could enhance particulate alkalinity [57]. Figure 5 illustrated the variations in acid and Figure S3 presented the correlation analysis between anion equivalents (AE) and cation equivalents (CE) in XY during pre-fireworks, fireworks and post-fireworks phases over three years. In 2023 and 2025, the acid showed the same dynamic patterns, enhanced from 0.70 and 0.73 pre-fireworks to 0.75 and 0.76 during fireworks, then to 0.84 and 0.79 post-fireworks, respectively. In 2024, the acid slightly increased from 0.78 pre-fireworks to 0.79 during fireworks, while it decreased to 0.74 post-fireworks. Similarly, Huang et al. (2019) confirmed a marked increase in acid influenced by massive firework activities in Xiamen [58]. Although the acid exhibited increasing trends during the fireworks period from 2023 to 2025, it remained below 1, indicating complete neutralization of anions and redundances of cations, leading to an alkaline aerosol state. This result demonstrated that CE might play a significant role in the atmospheric environment, aligned with reports of Xu et al. (2012) which also noted alkaline aerosol characteristics in Fuzhou [59]. Therefore, the persistent increases in aerosol acid in firework periods necessitated more stricter strategies on firework emissions during the Spring Festival in XY.

### 3.3. Nitrate and Sulfate Formation Mechanism

The nitrogen and sulfur oxidation ratios (NOR and SOR) served as key indicators for evaluating the formation of secondary inorganic aerosols [60]. The average values of NOR and SOR during the three stages were listed in Table 1, which were calculated as the mean of all valid hourly data points within each defined period. The NOR value ranged from 0.5 to 0.6 during the fireworks period in 2023–2024, which were 1.0–1.5 and 1.3–1.5 times higher than the pre-fireworks and post-fireworks periods. In 2025, NOR initially increased from 0.2 pre-fireworks to 0.4 during the fireworks stage, and subsequently to 0.5 post-fireworks. The SOR value varied from 0.6 to 0.8 during the fireworks period in 2023–2024, which were 1.0–1.2 and 1.1–1.2 times higher than the pre-fireworks and post-fireworks levels. In 2025, SOR increased significantly from 0.4 pre-fireworks to 0.7 during fireworks, then to 0.8 post-fireworks. The elevated NOR and SOR during fireworks clearly indicated enhanced NO_3_^−^ and SO_4_^2−^ formation.

**Table 1:** The variations in SOR, NOR, meteorological conditions and air pollutants during the pre-fireworks, fireworks and post-fireworks phases in XY over three years.

Parameters	2023			2024			2025		
	Pre	During	Post	Pre	During	Post	Pre	During	Post
SOR	0.5	0.6	0.5	0.8	0.8	0.7	0.4	0.7	0.8
NOR	0.4	0.6	0.4	0.5	0.5	0.4	0.2	0.4	0.5
RH	59.3	53.2	35.8	55.2	51.6	58.3	50.2	60.9	77.3
T	6.1	6.5	3.9	5.9	12.4	14.1	3.8	7.5	5.6
Ws	0.7	1.3	0.9	0.9	1.1	1.4	0.6	1.0	0.8
SO_2_	4.0	6.2	4.8	2.7	3.5	2.8	2.3	3.0	2.7
NO_2_	21.7	12.4	17.6	19.1	12.1	10.9	14.1	14.3	16.5
O_x_	62.4	86.4	67.1	121.4	100.5	74.8	60.3	72.2	52.2

**Figure 6:**
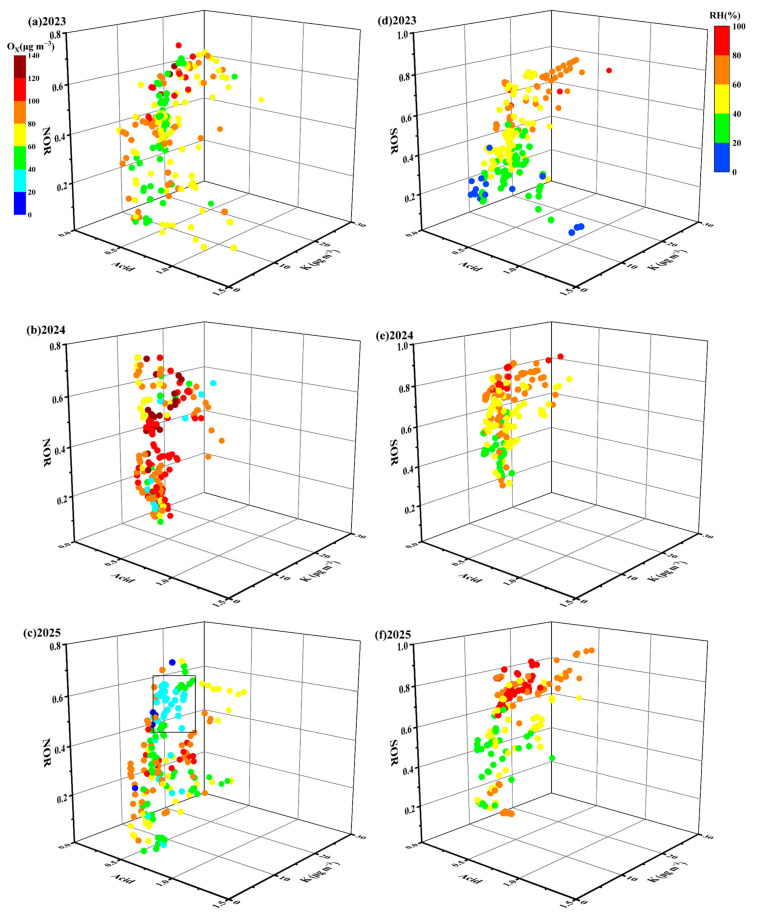
Relationship between NOR (SOR), O_x_ (RH), acid and K during the fireworks period in XY from 2023 to 2025: (**a**) NOR in 2023; (**b**) SOR in 2023; (**c**) NOR in 2024; (**d**) SOR in 2024; (**e**) NOR in 2025; (**f**) SOR in 2025. The rectangle in (**c**) represents several data points of high NOR, coupled with low O_x_ (≤45 μg m^−3^) but high RH (near 90.0%).

NOR showed positive correlations with O_x_ (R^2^ = 0.44–0.53, *p* < 0.01), acidity (R^2^ = 0.43–0.58, *p* < 0.01) and K (R^2^ = 0.31–0.45, *p* < 0.01) during fireworks period of 2023–2025 (Table S4). The strong association between NOR and O_x_ suggested that elevated O_x_ levels promoted the oxidation of NO_2_ to NO_3_^−^. Nitrate could be formed by reacting NO_2_ with OH radicals under high oxidative conditions via the gas-phase [61,62]. Figure S5 revealed a consistent increase trend in both NOR and O_x_ during fireworks of 2023–2025. The high NOR implied a significant enhancement in NO_3_^−^, primarily driven by the gas-phase with the elevated O_x_ concentrations. The persistent positive relationship between NOR and acidity indicated that the enhanced acidic conditions also favored NO_3_^−^ formation. Consequently, the combined effects of high O_x_ levels and enhanced acidity collectively drove the increase in NOR (Figure 6a,b). Nevertheless, high NOR was observed in Figure 6c, accompanied by low O_x_ (≤45 μg m^−3^) level but high RH (near 90.0%), indicating that nitrate was probably formed by the N_2_O_5_ hydrolysis through the heterogeneous processes under high-humidity conditions (Table S5, R^2^ = 0.80, *p* < 0.01) [63]. This observation aligned with prior study, which reported enhanced nitrate conversion under high RH despite low O_x_, likely due to heterogeneous processes [64]. Overall, these findings highlighted that the increase in NOR during firework events was driven by a combination of high O_x_, RH and acidity conditions via gas-phase and heterogeneous processes, respectively.

Significant positive correlations were observed between SOR and RH (R^2^ = 0.73–0.88, *p* < 0.01) acidity (R^2^ = 0.41–0.73, *p* < 0.01), and K (R^2^ = 0.54–0.85, *p* < 0.01) during fireworks in three years (Table S4). The good correlation between RH and SOR suggested that moist conditions were favorable for the conversion of SO_2_ to SO_4_^2−^. This finding was in agreement with Wang et al. (2021), who identified a sharp increase in SOR in Beijing when RH exceeded 40% [65]. The consistent growth trend between SOR and RH (Figure 1 and Figure S5) further revealed that SO_4_^2−^ formation was mainly facilitated by the aqueous-phase reaction [66]. Moreover, the positive relationship between SOR and acidity demonstrated that enhanced acidic conditions also promoted SO_4_^2−^ formation, which was supported by Fu et al. (2024) who found the increased production of SO_4_^2−^ under higher acidity [67]. Cheng et al. (2016) pointed out that elevated RH facilitated the rapid conversion of SO_2_ into particulate sulfate by neutralizing with NH_3_, which improved the particles’ acid and further promoted the sulfate formation [66]. Collectively, these results underscored a synergistic effect between high RH and elevated aerosol acidity in promoting SO_4_^2−^ production.

### 3.4. Source Apportionment of PM_2.5_

By using the Positive Matrix Factorization (PMF) monitoring model, six sources were identified in 2023 and 2025, including fireworks burning (FB), coal combustion (CC), vehicle emissions (VE), industrial emissions (IE), secondary aerosols (SA), and dust; five sources were analyzed in 2024, covering FB, CC, SA, dust and industrial emissions + vehicle emissions (IE + VE).

Figure S1 showed the source apportionment of PM_2.5_-bound elements resolved by the PMF model. Factor 1 was predominantly associated with OE, EC, Zn and Pb, which were typical tracers of vehicular emissions. Zn primarily originated from tire wear and brake abrasion [68]. Yan et al. (2022) found that the increasing numbers of motor vehicles in Shenzhen could significantly elevate Zn emission levels [69]. Although Pb has been phased out from gasoline in many regions, it might still derive from wear and tear of automotive components, such as brake pads and tires [70,71]. OC and EC were indicative of incomplete combustion processes, commonly associated with diesel and gasoline vehicles [72]. Factor 2 was characterized by high loadings of Cl^−^, OC and EC, attributed to coal combustion. Cl^−^ was mostly evolved from coal combustion, especially in domestic heating and power generation, while OC and EC were well-established markers of solid fuel combustion [73,74]. Factor 3 was defined by crustal elements, including Ca^2+^, Fe, Ti and Si. These four elements are commonly derived from construction activities and road dust, indicative of dust sources [75,76]. Factor 4 exhibited elevated levels of K^+^, Mg, Cu and Ba, highly associated with fireworks burning. K^+^ compounds served as key oxidizers, Mg enhanced brightness, Cu compounds produced blue light, and Ba was used for green flame coloration [77,78]. Factor 5 in 2023 and 2025 was dominated by SO_4_^2−^, NH_4_^+^ and NO_3_^−^, representing secondary aerosols’ source. The formation mechanism mainly involved the oxidation of SO_2_ and NO_x_, and the neutralization with NH_3_ [79]. However, factor 5 in 2024 was characterized by high loadings of Pb, Zn, Mn, Se, Cr, OC and EC. As mentioned earlier, Pb, Zn, OC and EC were characteristic elements of vehicle emissions. The high Mn, Se and Cr were typically emitted from ferrous metal smelting [80,81]. Therefore, this factor was identified as a mixed source, which was called the industrial emissions + vehicle emissions (IE + VE). Factor 6, heavily loaded by Se and Br, was indicative of industrial emissions. Se and Br were commonly released from coal-fired power plants, metal smelting, glass manufacturing and flame retardant processes [82].

**Figure 7:**
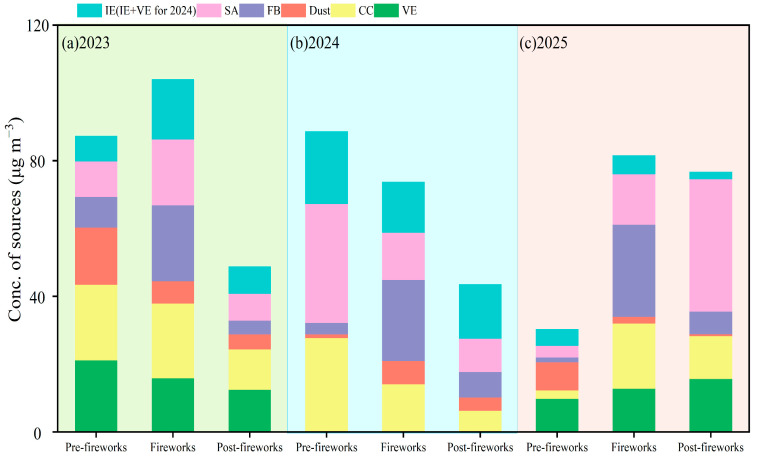
Concentration of emission sources to PM_2.5_ during pre-fireworks, fireworks and post-fireworks periods in XY from 2023 to 2025: (**a**) 2023; (**b**) 2024; (**c**) 2025.

The concentrations of each source were demonstrated in Figure 7. In 2023, the values of FB, SA and IE were 9.1, 10.5 and 7.4 μg m^−3^ pre-fireworks, greatly increasing by 147.4%, 85.4% and 138.4% during fireworks, while dropping by 82.1%, 59.3% and 54.5% post-fireworks, respectively. The sharp rise in FB was driven by widespread fireworks displays. The increase in IE reflected industrial recovery following the lifting of the COVID-19 control measures in 2023 [83]. SA growth was also influenced by firework emissions [84]. Similar variation in FB source was observed in Shandong, where FB contribution significantly increased from 16.0% during the benchmark period to 59.0% during the concentrated fireworks discharge periods [85]. VE, CC and dust exhibited similar patterns, decreasing their concentrations to PM_2.5_ from 21.2, 22.2 and 16.9 μg m^−3^ pre-fireworks to 15.8, 22.0 and 6.6 μg m^−3^ during fireworks, achieving 12.5, 11.9 and 4.5 μg m^−3^ post-fireworks. The reason for decreased VE was that large numbers of workers have returned home for the Spring Festival, while reduced dust levels resulted from diminished traffic and construction activities in the same period [86].

In 2024, the levels of FB and dust measured 19.9 and 5.7 μg m^−3^ during fireworks, 6.3–7.1 and 1.7–3.2 times higher than the values of the pre-fireworks and post-fireworks periods, respectively. In contrast, the concentrations of SA and CC dramatically declined from 29.2 and 23.2 μg m^−3^ to 11.6 to 11.8 μg m^−3^, and kept dropping to 8.2 and 5.3 μg m^−3^. The lower SA concentration during fireworks might be attributed to regional transport of secondary inorganic aerosols and favorable meteorological conditions [87], while the decreased CC was possibly due to elevated temperature (from 5.9 °C to 12.4 °C) during fireworks phase reducing residential coal consumption for heating. Meanwhile, the concentrations of IE + VE first declined from 17.7 μg m^−3^ to 12.4 μg m^−3^, and recovered to 13.3 μg m^−3^, reflecting population mobility for celebrating the Spring Festival.

In 2025, the levels of FB, CC and IE reached 27.2, 19.2 and 5.4 μg m^−3^ during fireworks, exceeding the pre-fireworks and post-fireworks values by factors of 1.1–19.3 and 1.5–4.1, respectively. The SA values showed a direct increasing trend, which were 3.4 μg m^−3^ pre-fireworks, 14.9 μg m^−3^ during fireworks and 39.1 μg m^−3^ post-fireworks, suggesting enhanced secondary formation. The level of VE initially increased from 9.8 to 12.9, and subsequently to 15.7 μg m^−3^. By contrast, dust values declined consistently, falling from 8.3 μg m^−3^ to 1.9 μg m^−3^, and further to 0.6 μg m^−3^. Overall, FB and SA collectively accounted for 40.2% to 51.6% of PM_2.5_ during the fireworks period from 2023 to 2025, which were the critical sources to the observed increase in PM_2.5_.

## 4. Conclusions

This study investigated the impacts of fireworks displays on PM_2.5_, chemical compositions, acidity and source contributions in a forestry city during the Spring Festival from 2023 to 2025. The results indicated that the fireworks period was characterized by significantly elevated levels of PM_2.5_, fireworks-related species (K, Cl^−^) and acidity. PM_2.5_/CO and SIA/CO ratios exhibited increasing trends during the fireworks period over three years, with factors of 1.4–2.3 and 1.1–3.4 related to levels of the pre-fireworks period, indicating obvious increases in secondary inorganic aerosols. Consequently, the increase in SOR (R^2^ = 0.73–0.88 for RH, R^2^ = 0.41–0.73 for acidity and R^2^ = 0.54–0.85 for K in three years, *p* < 0.01) and NOR (R^2^ = 0.44–0.53 for O_x_, R^2^ = 0.43–0.58 for acidity and R^2^ = 0.31–0.45 for K in three years, *p* < 0.01) during the fireworks period were amplified by the synergistic effects of high RH, elevated O_x_, and enhanced acidity, leading to more efficient SO_4_^2−^ and NO_3_^−^ formation. PMF method confirmed that the levels of fireworks burning (FB) and secondary aerosols (SA) source during fireworks rose by factors of 2.5–19.3 and 1.9–4.4, respectively, compared to the pre-fireworks phase. Therefore, this study emphasized the urgent need for adopting targeted policy interventions to inhibit fireworks combustion and secondary inorganic aerosols formation during the Spring Festival in future, given their promoting effects on increased PM_2.5_.

## Data Availability

The data presented in this study are available on request from the corresponding author.

## Supplementary Materials

The following supporting information can be downloaded at: https://www.mdpi.com/article/10.3390/toxics13121009/s1, Figure S1: Five and six factor profiles resolved by PMF in XY: (a) 2023; (b) 2024; (c) 2025; Figure S2: Correlation of observed PM2.5 concentration and reconstructed PM2.5 concentration resolved by PMF in XY; Figure S3: The relationships of AE with CE in XY.; Figure S4: The concentrations of NO2 and SO2 changes with increasing acidity in three years: (a) 2023; (b) 2024; (c) 2025; Figure S5: The SOR and NOR ratios and Ox concentrations from 2023 to 2025 in XY: (a) 2023; (b) 2024; (c) 2025; Table S1: The concentrations of PM2.5 and chemical compositions during the pre-fireworks, fireworks and post-fireworks periods in 2023. (unit: μg m^−3^ for PM2.5 and inorganic water-soluble ions while ng m^−3^ for trace elements); Table S2: The concentrations of PM2.5 and chemical compositions during the pre-fireworks, fireworks and post-fireworks periods in 2024. (unit: μg m^−3^ for PM2.5 and inorganic water-soluble ions while ng m^−3^ for trace elements); Table S3: The concentrations of PM2.5 and chemical compositions during the pre-fireworks, fireworks and post-fireworks periods in 2025. (unit: μg m^−3^ for PM2.5 and inorganic water-soluble ions while ng m^−3^ for trace elements); Table S4: Statistical analysis of NOR, SOR, RH, Ox, K and aerosol acid during three periods in 2023–2025; Table S5: Correlation analysis of SIA, Ws, RH and T during three periods in 2024; Table S6: Statistical analysis of PM2.5, SIA and CO in three years; Table S7: Correlation analysis of PM2.5, SIA and CO during three periods in 2023–2025. References [82,88,89,90,91,92,93] are cited in the Supplementary Materials.

## Abbreviations

The following abbreviations are used in this manuscript:

XY	Xinyang
FB	Fireworks burning
VE	Vehicle emissions
IE	Industrial emissions
IE + VE	Industrial emissions + Vehicle emissions
SA	Secondary aerosols
CC	Coal combustion

## References

[1] Fan S.D., Li Y., Liu C.F. (2021). Are Environmentally Friendly Fireworks Really “Green” for Air Quality? A Study from the 2019 National Day Fireworks Display in Shenzhen. Environ. Sci. Technol..

[2] He G.H., Cai M., Meng R.L., Hu J.X., Peng K., Hou Z.L., Zhou C.L., Xu X.J., Xiao Y.Z., Yu M. (2022). The Spring Festival Is Associated with Increased Mortality Risk in China: A Study Based on 285 Chinese Locations. Front. Med..

[3] Zhang Y.Y., Wei J.M., Tang A.H., Zheng A.H., Shao Z.X., Liu X.J. (2017). Chemical characteristics of PM_2.5_ during 2015 Spring Festival in Beijing, China. Aerosol Air Qual. Res..

[4] Seidel D.J., Birnbaum A.N. (2015). Effects of Independence Day fireworks on atmospheric concentrations of fine particulate matter in the United States. Atmos. Environ..

[5] Lin C.C. (2016). A review of the impact of fireworks on particulate matter in ambient air. J. Air Waste Manag. Assoc..

[6] Rai P., Furger M., Slowik J.G., Canonaco F., Fröhlich R., Hüglin C., Minguillón M.C., Patterson K., Baltensperger U., Prévôt A.S.H. (2020). Source apportionment of highly time-resolved elements during a firework episode from a rural freeway site in Switzerland. Atmos. Chem. Phys..

[7] Andradottir H.O., Thorsteinsson T. (2019). Repeated extreme particulate matter episodes due to fireworks in Iceland and stakeholders’ response. J. Clean. Prod..

[8] Drewnick F., Hings S.S., Curtius J., Eerdekens G., Williams J. (2006). Measurement of fine particulate and gas-phase species during the New Year’s fireworks 2005 in Mainz, Germany. Atmos. Environ..

[9] Cao X.Y., Zhang X.L., Tong D.Q., Chen W.W., Zhang S.C., Zhao H.M., Xiu A.J. (2018). Review on physicochemical properties of pollutants released from fireworks: Environmental and health effects and prevention. Environ. Rev..

[10] Hu B.X., Duan J.C., Liu S.J., Hu J.N., Zhang M., Kang P., Wang C. (2019). Evaluation of the effect of fireworks prohibition in the Beijing-Tianjin-Hebei and surrounding areas during the Spring Festival of 2018. Res. Environ. Sci..

[11] Song S.J., Gao M., Xu W.Q., Shao J.Y., Shi G.L., Wang S.X., Wang Y.X., Sun Y., McElroy M.B. (2018). Fine-particle pH for Beijing winter haze as inferred from different thermodynamic equilibrium models. Atmos. Chem. Phys..

[12] Ding J., Zhao P.S., Su J., Dong Q., Du X., Zhang Y.F. (2019). Aerosol pH and its driving factors in Beijing. Atmos. Chem. Phys..

[13] Wu C., Wang G.H., Wang J.Y., Li J.J., Ren Y.Q., Zhang L., Cao C., Li J., Ge S.S., Xie Y.N. (2018). Chemical characteristics of haze particles in Xi’an during Chinese Spring Festival: Impact of fireworks displays. J. Environ. Sci..

[14] Tian Y.Z., Wang J., Peng X., Shi G.L., Feng Y.C. (2014). Estimation of the direct and indirect impacts of fireworks on the physicochemical characteristics of atmospheric PM_10_ and PM_2.5_. Atmos. Chem. Phys..

[15] Foreback B., Dada L., Daellenbach K.R., Yan C., Wang L.L., Chu B.W., Zhou Y., Kokkonen T.V., Kurppa M., Pileci R.E. (2022). Measurement Report: A Multi-Year Study on the Impacts of Chinese New Year Celebrations on Air Quality in Beijing, China. Atmos. Chem. Phys..

[16] Chen S.Y., Jiang L.D., Liu W.L., Song H. (2022). Fireworks Regulation, Air Pollution, and Public Health: Evidence from China. Reg. Sci. Urban Econ..

[17] Zhang X.X., Gu X.C., Cheng C.X., Yang D.Y. (2020). Spatiotemporal heterogeneity of PM_2.5_ and its relationship with urbanization in North China from 2000 to 2017. Sci. Total. Environ..

[18] Liu X.Y., Zhao C.M., Shen X.Z., Jin T. (2022). Spatiotemporal variations and sources of PM_2.5_ in the Central Plains Urban Agglomeration, China. Air Qual. Atmos. Health.

[19] (2018). Technical Specifications for Operation and Quality Control of Ambient Air Quality Automated Monitoring System for Particulate Matter (PM10 and PM2.5).

[20] (2018). Gas Analysis—Guide for Quality Assurance of On-Line Automatic Measuring System.

[21] Liu B.S., Yang J.M., Yuan J., Dai Q.L., Li T.K., Bi X.H., Feng Y.C., Xiao Z.M., Zhang Y.F., Xu H. (2017). Source apportionment of atmospheric pollutants based on the online data by using PMF and ME2 models at a megacity, China. Atmos. Res..

[22] (2015). Field-Calibration Method for Data Logger of Automatic Weather Station.

[23] Qiao B.Q., Chen Y., Tian M., Wang H.B., Yang F.M., Shi G.M., Zhang L.M., Peng C., Luo Q., Ding S.M. (2019). Characterization of water soluble inorganic ions and their evolution processes during PM_2.5_ pollution episodes in a small city in southwest China. Sci. Total Environ..

[24] He K., Zhao Q., Ma Y., Duan F., Yang F., Shi Z., Chen G. (2012). Spatial and seasonal variability of PM_2.5_ acidity at two Chinese megacities: Insights into the formation of secondary inorganic aerosols. Atmos. Chem. Phys..

[25] Zhou H.J., Lü C.W., He J., Gao M.S., Zhao B.Y., Ren L.M., Zhang L.J., Fan Q.Y., Liu T., He Z.X. (2018). Stoichiometry of water-soluble ions in PM_2.5_: Application in source apportionment for a typical industrial city in semi-arid region, Northwest China. Atmos. Res..

[26] Paatero P., Tapper U. (1994). Positive matrix factorization: A non-negative factor model with optimal utilization of error estimates of data values. Environmetrics.

[27] Lucarelli F., Calzolai G., Chiari M., Giardi F., Czelusniak C., Nava S. (2020). Hourly Elemental Composition and Source Identification by Positive Matrix Factorization (PMF) of Fine and Coarse Particulate Matter in the High Polluted Industrial Area of Taranto (Italy). Atmosphere.

[28] Li J.W., Ren L.H., Wu Y.F., Zhang R.J., Yang X.Y., Li G., Gao E.H., An J.T., Xu Y.S. (2024). Different variations in PM_2.5_ sources and their specific health risks in different periods in a heavily polluted area of the Beijing-Tianjin-Hebei region of China. Atmos. Res..

[29] Shi G.L., Liu G.R., Tian Y.Z., Zhou X.Y., Peng X., Feng Y.C. (2014). Chemical characteristic and toxicity assessment of particle associated PAHs for the short-term anthropogenic activity event: During the Chinese New Year’s Festival in 2013. Sci. Total Environ..

[30] Ye C., Chen R.S., Chen M.X. (2015). The impacts of Chinese Nian culture on air pollution. J. Clean. Prod..

[31] Liu J., Man Y., Liu Y. (2014). Temporal variability of PM_10_ and PM_2.5_ inside and outside a residential home during 2014 Chinese Spring Festival in Zhengzhou, China. Nat. Hazards.

[32] Huang K., Zhuang G., Lin Y., Wang Q., Fu J.S., Zhang R., Li J., Deng C., Fu Q. (2012). Impact of anthropogenic emission on air quality over a megacity-revealed from an intensive atmospheric campaign during the Chinese Spring Festival. Atmos. Chem. Phys..

[33] Li L., Li Q., Huang L., Wang Q., Zhu A.S., Xu J., Liu Z.Y., Li H.L., Shi L.S., Li R. (2020). Air quality changes during the COVID-19 lockdown over the Yangtze River Delta Region: An insight into the impact of human activity pattern changes on air pollution variation. Sci. Total Environ..

[34] Zhou X.T., Strezov V., Jiang Y.J., Kan T., Evans T. (2022). Temporal and spatial variations of air pollution across China from 2015 to 2018. J. Environ. Sci..

[35] Song M.D., Liu X.G., Tan Q.W., Feng M., Qu Y., An J.L., Zhang Y.H. (2019). Characteristics and formation mechanism of persistent extreme haze pollution events in Chengdu, southwestern China. Environ. Pollut..

[36] Dai Q.L., Ding J., Hou L.L., Li L.X., Cai Z.Y., Liu B.S., Song C.B., Bi X.H., Wu J.H., Zhang Y.F. (2021). Haze episodes before and during the COVID-19 shutdown in Tianjin, China: Contribution of fireworks and residential burning. Environ. Pollut..

[37] Ambade B. (2018). The air pollution during Diwali festival by the burning of fireworks in Jamshedpur city. India. Urban Clim..

[38] Tanda S., Ličbinský R., Hegrová J., Goessler W. (2019). Impact of NewYear’s Eve Fireworks on the Size Resolved Element Distributions in Airborne Particles. Environ. Int..

[39] Moreno T., Querol X., Alastuey A., Amato F., Pey J., Pandolfi M., Kuenzli N., Bouso L., Rivera M., Gibbons W. (2010). Effect of fireworks events on urban background trace metal aerosol concentrations: Is the cocktail worth the show?. J. Hazard. Mater..

[40] Zhang J.M., Yang L.X., Chen J.M., Mellouki A., Jiang P., Gao Y., Li Y.Y., Yang Y.M., Wang W.X. (2017). Influence of fireworks displays on the chemical characteristics of PM_2.5_ in rural and suburban areas in Central and East China. Sci. Total Environ..

[41] Zhao N., Wang G., Zhu Z.Y., Liu Z.L., Tian G.M., Liu Y.Q., Gao W.K., Lang J.L. (2023). Impact of fireworks burning on air quality during the Spring Festival in 2021–2022 in Linyi, a central city in the North China Plain. Environ. Sci. Pollut. Res..

[42] Yi Y.N., Li Q., Zhang K., Li R., Yang L.M., Liu Z.Q., Zhang X.J., Wang S.Y., Wang Y.J., Chen H. (2022). Highly time-resolved measurements of elements in PM_2.5_ in Changzhou, China: Temporal variation, source identification and health risks. Sci. Total Environ..

[43] Shang X.H., Wang S.B., Zhang R.Q., Yuan M.H., Xu Y.F., Ying Q. (2024). Variations of the source-specific health risks from elements in PM_2.5_ from 2018 to 2021 in a Chinese megacity. Atmos. Pollut. Res..

[44] Zhang J.Z., Zhou X.H., Wang Z., Yang L.X., Wang J., Wang W.X. (2018). Trace elements in PM_2.5_ in Shandong Province: Source identification and health risk assessment. Sci. Total Environ..

[45] Hasanbeigi A., Lobscheid A., Lu H.Y., Price L., Dai Y. (2013). Quantifying the co-benefits of energy-efficiency policies: A case study of the cement industry in Shandong Province, China. Sci. Total Environ..

[46] Feng J.L., Yu H., Su X.F., Liu S.H., Li Y., Pan Y.P., Sun J.H. (2016). Chemical composition and source apportionment of PM_2.5_ during Chinese Spring Festival at Xinxiang, a heavily polluted city in North China: Fireworks and health risks. Atmos. Res..

[47] Du X.X., Shi G.M., Zhao T.L., Yang F.M., Zheng X.B., Zhang Y.J., Tan Q.W. (2020). Contribution of secondary particles to wintertime PM_2.5_ during 2015–2018 in a major urban area of the Sichuan Basin, Southwest China. Earth Space Sci..

[48] He J.J., Gong S.L., Yu Y., Yu L.J., Wu L., Mao H.J., Song C.B., Zhao S.P., Liu H.L., Li X.Y. (2017). Air pollution characteristics and their relation to meteorological conditions during 2014–2015 in major Chinese cities. Environ. Pollut..

[49] Zhang Y.L., Cao F. (2015). Fine particulate matter (PM_2.5_) in China at a city level. Sci. Rep..

[50] Liu X.S., Hadiatullah H., Tai P.F., Xu Y.L., Zhang X., Schnelle-Kreis J., Schloter-Hai B., Zimmermann R. (2021). Air pollution in Germany: Spatio-temporal variations and their driving factors based on continuous data from 2008 to 2018. Environ. Pollut..

[51] Song C.B., Wu L., Xie Y.C., He J.J., Chen X., Wang T., Lin Y.C., Jin T.S., Wang A.X., Liu Y. (2017). Air pollution in China: Status and spatiotemporal variations. Environ. Pollut..

[52] Pongpiachan S., Iijima A., Cao J.J. (2018). Hazard Quotients, Hazard Indexes, and Cancer Risks of Toxic Metals in PM10 during Firework Displays. Atmosphere.

[53] Huang R.J., Zhang Y.L., Bozzetti C., Ho K.F., Cao J.J., Han Y.M., Daellenbach K.R., Slowik J.G., Platt S.M., Canonaco F. (2014). High secondary aerosol contribution to particulate pollution during haze events in China. Nature.

[54] Blitz M.A., Hughes K.J., Pilling M.J. (2003). Determination of the high-pressure limiting rate coefficient and the enthalpy of reaction for OH+SO_2_. J. Phys. Chem..

[55] Cai W.J., Liao H., Wang H.J., Wu L.X. (2017). Weather conditions conducive to Beijing severe haze more frequent under climate change. Nat. Clim. Change.

[56] Zhou J.B., Xing Z.Y., Deng J.J., Du K. (2016). Characterizing and sourcing ambient PM_2.5_ over key emission regions in China I: Water-soluble ions and carbonaceous fractions. Atmos. Environ..

[57] Yang Y.J., Zhou R., Wu J.J., Yu Y., Ma Z.Q., Zhang L.J., Di Y.A. (2015). Seasonal variations and size distributions of water-soluble ions in atmospheric aerosols in Beijing, 2012. J. Environ. Sci..

[58] Wang S.S., Yu R.L., Shen H.Z., Wang S., Hu Q.C., Cui J.Y., Yan Y., Huang H.B., Hu G.R. (2019). Chemical characteristics, sources, and formation mechanisms of PM_2.5_ before and during the Spring Festival in a coastal city in Southeast China. Environ. Pollut..

[59] Xu L.L., Chen X.Q., Chen J.S., Zhang F.W., He C., Zhao J.P., Yin L.Q. (2012). Seasonal variations and chemical compositions of PM_2.5_ aerosol in the urban area of Fuzhou, China. Atmos. Res..

[60] Huang R.J., Duan J., Li Y.J., Chen Q., Chen Y., Tang M.J., Yang L., Ni H.Y., Lin C.S., Xi W. (2020). Effects of NH_3_ and alkaline metals on the formation of particulate sulfate and nitrate in wintertime Beijing. Sci. Total Environ..

[61] Bertram T.H., Thornton J.A. (2009). Toward a general parameterization of N_2_O_5_ reactivity on aqueous particles: The competing effects of particle liquid water, nitrate and chloride. Atmos. Chem. Phys..

[62] Yao L., Fan X.L., Yan C., Kurtén T., Daellenbach K.R., Li C., Wang Y.H., Guo Y.S., Dada L., Rissanen M.P. (2020). Unprecedented Ambient Sulfur Trioxide (SO_3_) Detection: Possible Formation Mechanism and Atmospheric Implications. Environ. Sci. Technol. Lett..

[63] Wagner N., Riedel T., Young C., Bahreini R., Brock C., Dubé W., Kim S., Middlebrook A., Öztürk F., Roberts J. (2013). N_2_O_5_ uptake coefficients and nocturnal NO_2_ removal rates determined from ambient wintertime measurements. J. Geophys. Res. Atmos..

[64] McDuffie E.E., Womack C.C., Fibiger D.L., Dube W.P., Franchin A., Middlebrook A.M., Goldberger L., Lee B.H., Thornton J.A., Moravek A. (2019). On the contribution of nocturnal heterogeneous reactive nitrogen chemistry to particulate matter formation during wintertime pollution events in Northern Utah. Atmos. Chem. Phys..

[65] Wang Y.Y., Li Z.Q., Wang Q.Y., Jin X.A., Yan P., Cribb M., Li Y.N., Yuan C., Wu H., Ren R.M. (2021). Enhancement of secondary aerosol formation by reduced anthropogenic emissions during Spring Festival 2019 and enlightenment for regional PM_2.5_ control in Beijing. Atmos. Chem. Phys..

[66] Cheng Y.F., Zheng G.J., Wei C., Mu Q., Zheng B., Wang Z.B., Gao M., Zhang Q., He K.B., Carmichael G. (2016). Reactive nitrogen chemistry in aerosol water as a source of sulfate during haze events in China. Sci. Adv..

[67] Fu X.X., Wang X.M., Liu T.Y., He Q.F., Zhang Z., Zhang Y.L., Song W., Dai Q.W., Chen S., Dong F.Q. (2024). Secondary inorganic aerosols and aerosol acidity at different PM_2.5_ pollution levels during winter haze episodes in the Sichuan Basin, China. Sci. Total Environ..

[68] Huang X.F., Zou B.B., He L.Y., Hu M., Prévôt A.S.H., Zhang Y.H. (2018). Exploration of PM_2.5_ sources on the regional scale in the Pearl River Delta based on ME-2 modeling. Atmos. Chem. Phys..

[69] Yan R., Peng X., Lin W., He L., Wei F., Tang M., Huang X. (2022). Trends and challenges regarding the source-specific health risk of PM_2.5_-bound metals in a Chinese megacity from 2014 to 2020. Environ. Sci. Technol..

[70] Kleeman M.J., Schauer J.J., Cass G.R. (2000). Size and composition distribution of fine particulate matter emitted from motor vehicles. Environ. Sci. Technol..

[71] Meng F.L., Liu D.X., Bu T.X., Zhang M.Y., Peng J., Ma J.H. (2025). Assessment of pollution and health risks from exposure to heavy metals in soil, wheat grains, drinking water, and atmospheric particulate matter. J. Environ. Manag..

[72] He L.Y., Huang X.F., Xue L., Hu M., Lin Y., Zheng J., Zhang R.Y., Zhang Y.H. (2011). Submicron aerosol analysis and organic source apportionment in an urban atmosphere in Pearl River Delta of China using high-resolution aerosol mass spectrometry. J. Geophys. Res..

[73] Liu Y.Y., Xing J., Wang S.X., Fu X., Zheng H.T. (2018). Source-specific speciation profiles of PM_2.5_ for heavy metals and their anthropogenic emissions in China. Environ. Pollut..

[74] Park J.A., Kim H., Kim Y., Heo J., Kim S.W., Jeon K., Yi S.M., Hopke P.K. (2022). Source apportionment of PM_2.5_ in Seoul, South Korea and Beijing, China using dispersion normalized PMF. Sci. Total Environ..

[75] Nguyen Q.T., Skov H., Sorensen L.L., Jensen B.J., Grube A.G., Massling A., Glasius M., Nojgaard J.K. (2013). Source apportionment of particles at station Nord, north East Greenland during 2008–2010 using COPREM and PMF analysis. Atmos. Chem. Phys..

[76] Men C., Liu R.M., Xu F., Wang Q.R., Guo L.J., Shen Z.Y. (2018). Pollution characteristics, risk assessment, and source apportionment of heavy metals in road dust in Beijing, China. Sci. Total Environ..

[77] Sijimol M.R., Mohan M. (2014). Environmental impacts of perchlorate with special reference to fireworks-a review. Environ. Monit. Assess..

[78] Yu J.Y., Chen L.P., Peng J.H. (2012). Thermal hazard research of smokeless fireworks. J. Therm. Anal. Calorim..

[79] Martín-Alberca C., García-Ruiz C. (2014). Analytical techniques for the analysis of consumer fireworks. TrAC Trends Anal. Chem..

[80] Chen H., Yan Y.L., Hu D.M., Peng L., Wang C. (2024). Chang PM_2.5_-bound heavy metals in a typical industrial city of Changzhi in North China: Pollution sources and health risk assessment. Atmos. Environ..

[81] Yang X., Zheng M., Liu Y., Yan C.Q., Liu J.Y., Liu J.M., Cheng Y. (2022). Exploring sources and health risks of metals in Beijing PM_2.5_: Insights from long-term online measurements. Sci. Total Environ..

[82] Chang Y., Kan H., Xie M., Deng C., Zou Z., Liu S., Zhang Y. (2018). First long-term and near real-time measurement of trace elements in China’s urban atmosphere: Temporal variability, source apportionment and precipitation effect. Atmos. Chem. Phys..

[83] Kong L., Tang X., Zhu J., Wang Z.F., Sun Y.L., Fu P.Q., Gao M., Wu H.J., Lu M.M., Wu Q. (2023). Unbalanced emission reductions of different species and sectors in China during COVID-19 lockdown derived by multi-species surface observation assimilation. Atmos. Chem. Phys..

[84] Zhang Y.J., Li W.S., Li L., Li M., Zhou Z., Yu J.Z., Zhou Y. (2024). Source Apportionment of PM_2.5_ Using PMF Combined Online Bulk and Single-Particle Measurements: Contribution of Fireworks and Biomass Burning. J. Environ. Sci..

[85] Xie J., Wang G.X., Bi Y.L., Ding C., Qiao J., Wang L.M., Wang C.W., Qiu X.G. (2025). Impacts of fireworks on urban air quality during Spring Festivals of 2022–2024 in Shandong Province, China. Environ. Monit. Assess..

[86] Chen S.Y., Zhang X.R., Lin J.T., Huang J.P., Zhao D., Yuan T.G., Huang K.N., Luo Y., Zang Z., Qiu Y.A. (2019). Fugitive Road Dust PM_2.5_ Emissions and Their Potential Health Impacts. Environ. Sci. Technol..

[87] Wang H.L., Qiao L.P., Lou S.R., Zhou M., Chen J.M., Wang Q., Tao S.K., Chen C.H., Huang H.Y., Li L. (2015). PM_2.5_ pollution episode and its contributors from 2011 to 2013 in urban Shanghai, China. Atmos. Environ..

[88] Norris G., Duvall R., Brown S., Bai S. (2014). EPA Positive Matrix Factorization (PMF) 5.0 Fundamentals and User Guide.

[89] Amato F., Hopke P.K. (2012). Source apportionment of the ambient PM_2.5_ acrossst. Louis using constrained positive matrix factorisation. Atmos. Environ..

[90] Hopke P.K. (2016). Review of receptor modeling methods for source apportionment. J. Air Waste Manag. Assoc..

[91] Li H., Wu H., Wang Q., Yang M., Li F., Sun Y., Qian X., Wang J., Wang C. (2017). Chemical partitioning of fine particle-bound metals on haze-fog and non-haze-fog days in Nanjing, China and its contribution to human health risks. Atmos. Res..

[92] Huang R., Cheng R., Jing M., Yang L., Li Y., Chen Q., Chen Y., Yan J., Lin C., Wu Y. (2018). Source-specific health risk analysis on particulate trace elements: Coal combustion and traffic emission as major contributors in wintertime Beijing. Environ. Sci. Technol..

[93] Lin Y., Zhang Y., Song W., Yang X., Fan M. (2020). Specific sources of health risks caused by size-resolved PM-bound metals in a typical coal-burning city of northern China during the winter haze event. Sci. Total Environ..

