# Supplementary material for: Increased PM2.5 Caused by Enhanced Fireworks Burning and Secondary Aerosols in a Forested City of North China During the 2023–2025 Spring Festivals
Source: Toxics. 2025 Nov 21;13(12):1009. doi: 10.3390/toxics13121009 (PMC12736846; doi:10.3390/toxics13121009)
Supplement: Supplementary file 1 [file toxics-13-01009-s001.zip › toxics-3959598-supplementary.pdf]

Increased $PM_{2.5}$ caused by enhanced fireworks burning and secondary aerosols in a Forested City of the North China During the 2023–2025 Spring Festivals

Supplementary text: the Positive Matrix Factorization (PMF) model

Determining potential emission sources was an essential step for implementing air pollution strategies [1]. We adopted the PMF model (US EPA 5.0) to identify potential $PM_{2.5}$ sources based on the concentrations and uncertainties of 22 species (NH_4^+, NO_3^-, SO_4^{2-}, Ca^{2+}, K^+, Mg^{2+}, Cl^-, OC, EC, Fe, Pb, Zn, Mn, Se, Cr, Cu, Ti, Ba, As, Br, Cs and Si) and the $PM_{2.5}$ mass concentrations.

In estimating the uncertainty of input components, values were replaced by half of the MDL when their concentrations below or equal to the MDL, with the corresponding uncertainty set at 5/6 of the MDL [2]. An uncertainty of 20% was assigned for concentrations of elements above the method detection limit (MDL) [3]. Missing data were substituted with the median concentration, and their uncertainty was defined as four times the uncertainty of the measured values [4]. The uncertainty (UNC) was calculated as follows:

$$\text{UNC}=\frac{5}{6}\text{MDL} \tag{S1}$$

$$\text{UNC}=\sqrt{(\text{error rate} \times \text{mass concentration})^2+(\text{MDL})^2} \tag{S2}$$

Based on the PMF model standards, species classification is determined by using the signal-to-noise ratio (S/N) values. If S/N > 1, the species was labeled as “Strong”. If the S/N ratio was between 0.5 and 1, the species was classified as “Weak”, and if S/N < 0.5, the species was categorized as “Bad” [5,6].

Bootstrap (BS), displacement (DISP), and bootstrapping with displacement (BS-DISP) were also performed to analyze the uncertainty of the PMF model [7]. Moreover, the robust correlation between observed and reconstructed $PM_{2.5}$ confirmed the validity of our results (Figure S2).

Finally, six sources were identified in 2023 and 2025, including fireworks burning (FB), coal combustion (CC), vehicle emissions (VE), industrial emissions (IE), secondary aerosols (SA), and Dust; Five sources were analyzed in 2024, including FB, CC, SA, Dust and industrial emissions + vehicle emissions (IE+VE). The 5 and 6 factors solution is shown in Figure S1, respectively.

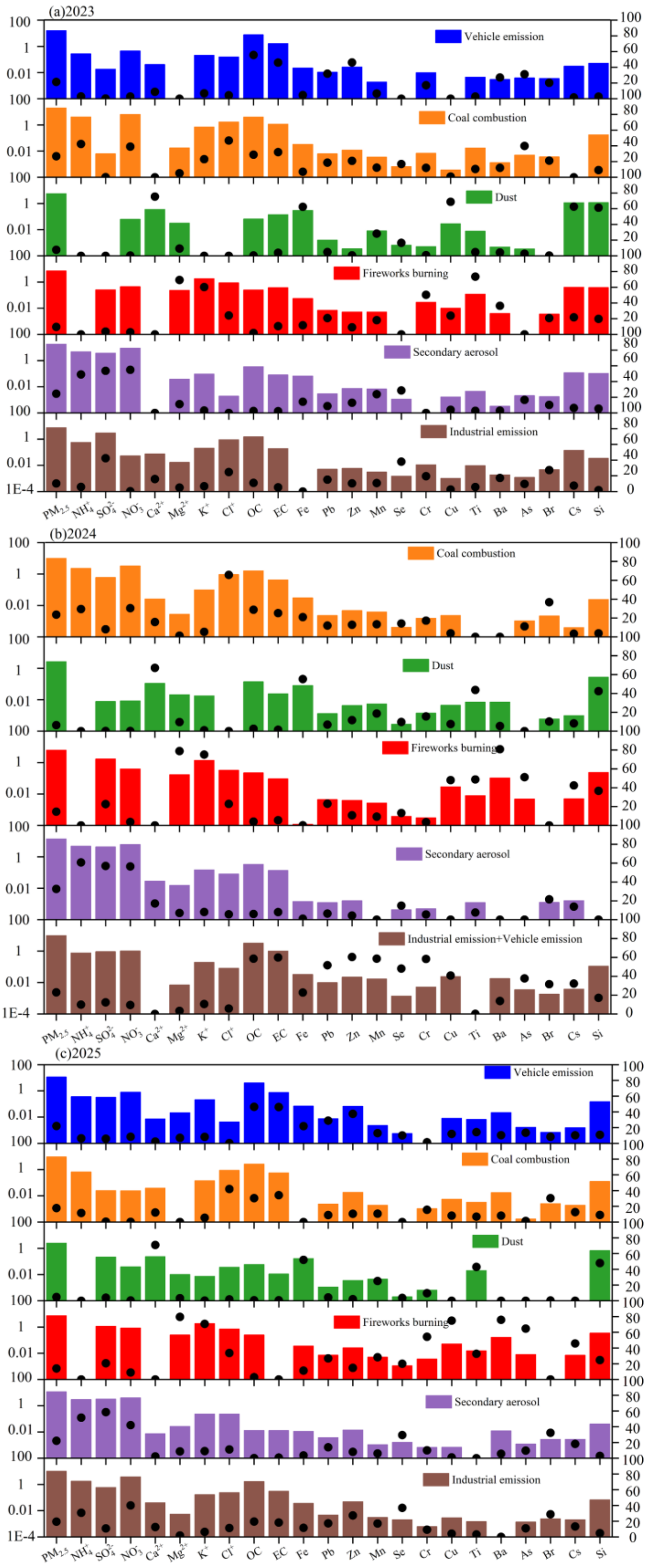

Figure S1. Five and six factor profiles resolved by PMF in XY: (**a**) 2023; (**b**) 2024; (**c**) 2025.

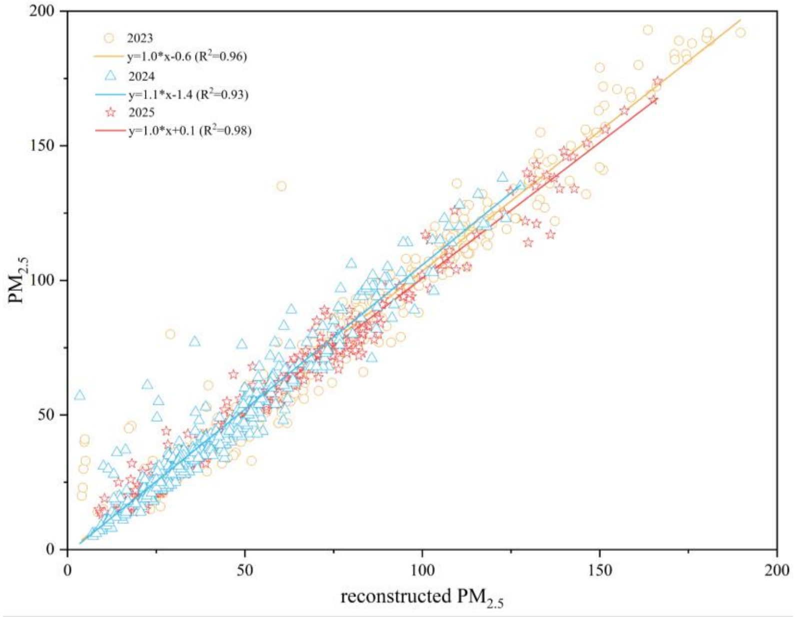

Figure S2. Correlation of observed $PM_{2.5}$ concentration and reconstructed $PM_{2.5}$ concentration resolved by PMF in XY.

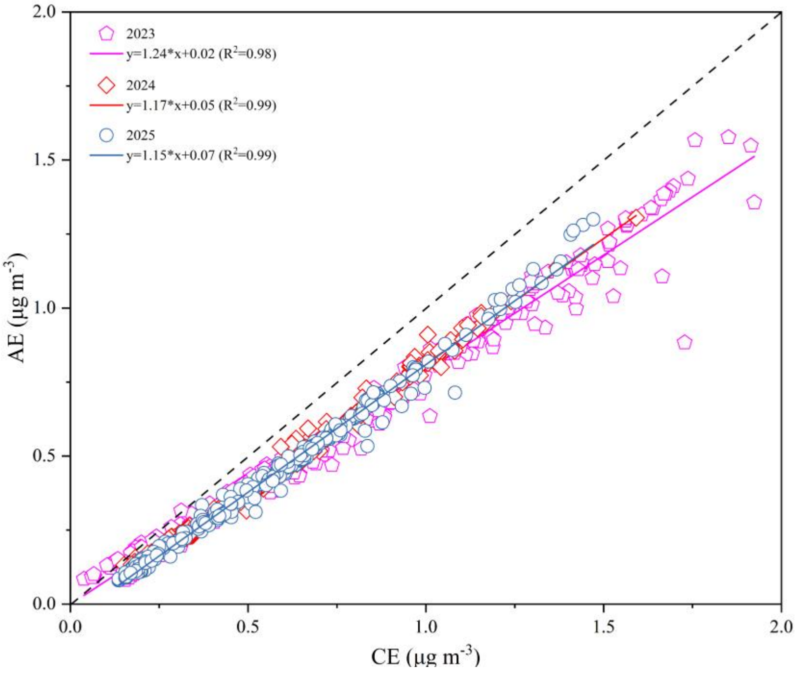

Figure S3. The relationships of AE with CE in XY.

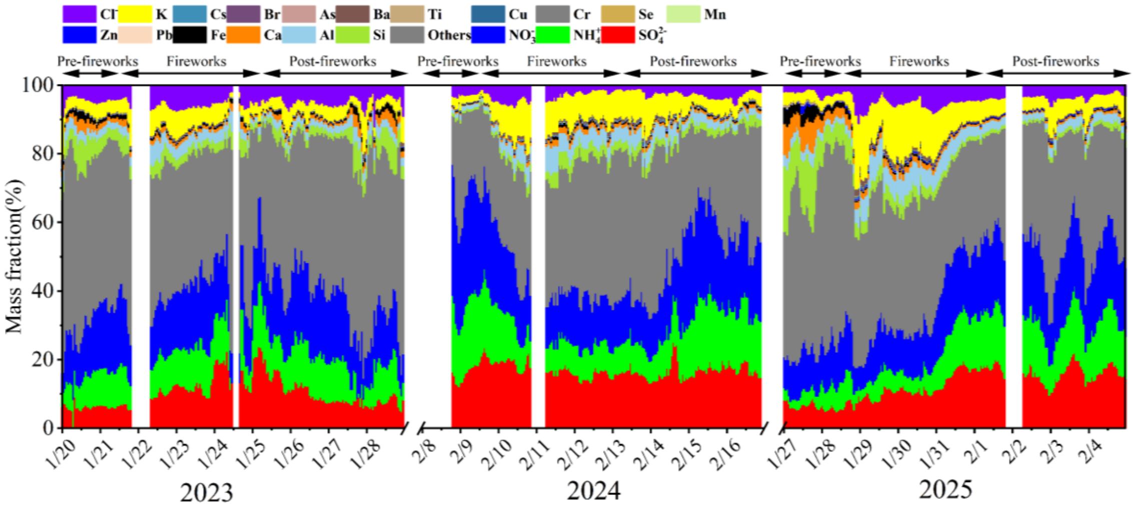

Figure S4. The concentrations of NO_2 and SO_2 changes with increasing acidity in three years: (**a**) 2023; (**b**) 2024; (**c**) 2025.

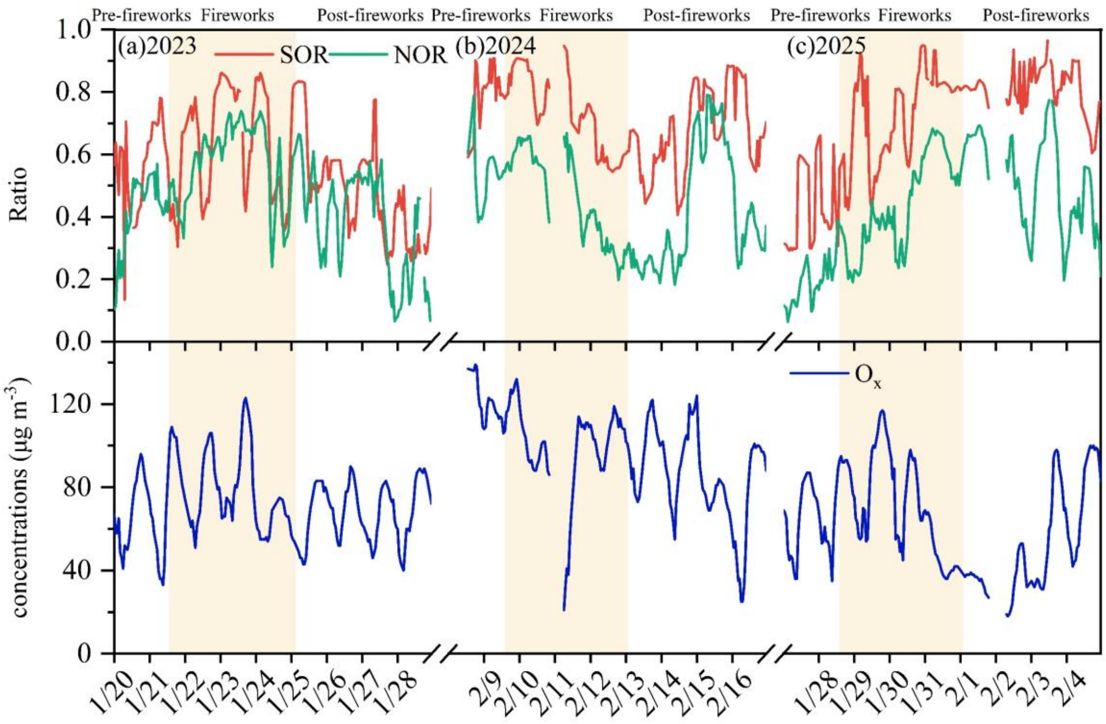

Figure S5. The SOR and NOR ratios and O_x concentrations from 2023 to 2025 in XY: (**a**) 2023; (**b**) 2024; (**c**) 2025.

Tables S1. The concentrations of $PM_{2.5}$ and chemical compositions during the pre-fireworks, fireworks and post-fireworks periods in 2023. (unit: μg m^{-3} for $PM_{2.5}$, inorganic water-soluble ions and air pollutants, while ng m^{-3} for trace elements)

	Pre-fireworks	fireworks	Post-fireworks
$PM_{2.5}$	87.9±27.7	113.6±53.5	50.4±23.3
Cl^-	4.2±2.4	7.2±4.3	2.7±1.7
SO_4^{2-}	5.3±2.2	12.7±7.0	5.0±2.5
NO_3^-	14.0±6.6	19.3±10.1	8.8±5.2
NH_4^+	7.9±3.6	12.3±6.4	5.5±3.2
K	3397.2±3025.4	7648.7±4816.5	2123.9±1650.0
Ca	1593.6±473.6	1355.5±563.5	588.2±219.5
Fe	1076.4±266.2	648.1±257.4	338.6±135.2
Pb	46.8±39.5	58.2±31.9	26.9±16.6
Zn	66.4±35.2	82.3±41.2	41.8±27.6
Mn	47.4±16.7	47.9±20.7	21.5±13.0
Se	4.1±1.9	5.2±2.9	2.9±1.8
Cr	0.1±0.5	0.2±0.7	0.7±1.6
Cu	78.7±76.9	133.3±84.0	48.0±41.3
Ti	104.4±35.0	70.2±29.7	31.7±18.8
Ba	270.4±397.4	456.1±319.5	136.1±196.5
As	11.3±10.9	22.5±14.9	8.3±6.5
Br	11.9±6.4	15.4±7.8	9.8±6.5
Cs	17.3±11.3	33.4±18.7	11.8±7.2
Si	4284.4±1201.7	3441.2±1393.0	1348.2±458.6
Al	2248.7±580.8	4346.9±2424.3	1137.7±470.7
Total elements	13857.1	18365.2	5876.3

Tables S2. The concentrations of $PM_{2.5}$ and chemical compositions during the pre-fireworks, fireworks and post-fireworks periods in 2024. (unit: μg m^{-3} for $PM_{2.5}$, inorganic water-soluble ions and air pollutants, while ng m^{-3} for trace elements)

	Pre-fireworks	fireworks	Post-fireworks
$PM_{2.5}$	59.9±8.4	66.2±25.5	36.2±19.4
Cl^{-}	1.7±0.5	2.5±1.9	1.0±0.7
SO_4^{2}	9.1±2.3	11.9±5.3	5.9±2.7
NO_3^{-}	18.8±4.7	11.9±6.0	5.9±2.7
NH_4^{+}	11.4±5.7	7.8±4.5	5.0±3.1
K	1350.4±546.4	5602.2±3325.8	2094.3±1355.3
Ca	172.2±60.6	643.2±276.6	304.1±162.2
Fe	123.1±31.1	234.9±136.4	156.8±65.2
Pb	21.4±4.6	33.2±10.8	22.8±12.9
Zn	45.2±14.9	59.1±17.8	41.2±19.6
Mn	33.4±13.3	50.5±16.7	35.4±13.5
Se	3.8±1.0	4.3±1.5	3.1±1.8
Cr	10.3±5.4	14.9±6.6	10.3±6.1
Cu	44.6±26.6	142.3±74.6	70.0±42.4
Ti	6.1±3.8	39.5±17.8	18.4±12.1
Ba	70.8±50.4	395.5±260.9	145.0±107.1
As	5.8±2.3	20.1±12.3	9.8±8.1
Br	9.9±1.8	6.8±3.3	6.6±3.2
Cs	11.4±2.9	25.9±12.0	13.4±8.1
Si	400.5±115.8	1587.6±661.8	753.1±330.6
Al	815.3±125.7	2788.2±1680.9	1097.2±494.9
Total elements	3123.8	11648.0	4781.6

Tables S3. The concentrations of $PM_{2.5}$, chemical compositions, meteorological conditions and air pollutants during the pre-fireworks, fireworks and post-fireworks periods in 2025. (unit: μg m^{-3} for $PM_{2.5}$, inorganic water-soluble ions and air pollutants, while ng m^{-3} for trace elements)

	Pre-fireworks	fireworks	Post-fireworks
$PM_{2.5}$	24.9±7.3	81.2±35.8	77.8±27.9
Cl^-	0.6±0.4	4.9±3.5	2.8±1.4
SO_4^{2}	1.5±0.3	9.5±4.6	12.7±5.2
NO_3^-	2.9±1.0	11.2±5.9	16.2±6.0
NH_4^+	1.2±0.7	6.3±4.1	11.6±3.9
K	690.4±137.4	10157.0±6305.7	3960.0±2133.2
Ca	1220.3±255.1	1201.0±586.4	475.6±221.3
Fe	671.1±135.7	361.5±137.3	211.0±91.6
Pb	8.5±4.0	47.3±28.1	35.6±16.5
Zn	76.9±114.9	254.8±120.0	195.8±75.7
Mn	14.8±10.1	29.1±21.5	16.4±12.5
Se	1.9±1.4	8.1±3.5	7.9±3.0
Cr	2.7±3.3	17.4±13.9	7.5±5.9
Cu	8.3±11.1	210.1±165.9	73.2±56.0
Ti	74.7±15.2	91.9±56.9	34.2±21.7
Ba	38.8±14.1	693.6±533.7	263.5±188.5
As	0.7±1.0	33.4±26.0	14.6±9.7
Br	0.6±0.9	11.9±9.1	14.2±9.2
Cs	2.0±1.9	38.1±20.9	19.9±9.6
Si	2515.1±527.3	2377.3±1025.3	990.7±403.6
Al	587.1±48.4	4580.4±2293.0	1891.1±745.0
Total elements	5914.0	20112.7	8211.3

Table S4. Statistical analysis of NOR, SOR, RH, O_x, K and aerosol acid during three periods in 2023-2025.

		Pre-fireworks		Fireworks		Post-fireworks	
		NOR	SOR	NOR	SOR	NOR	SOR
2023	RH	-0.17	0.78**	0.34	0.73**	0.30**	0.62**
	Ox	0.38**	-0.42**	0.53**	-0.66**	0.58	-0.55**
	Acid	0.16	0.91**	0.43**	0.73**	-0.69	-0.38
	K	0.38**	0.43**	0.31**	0.54**	0.37	0.36**
2024	RH	0.64**	0.67**	0.70**	0.81**	0.33**	0.72**
	Ox	0.22	-0.45**	0.47**	-0.18	0.27	-0.55**
	Acid	0.28	0.14	0.58**	0.48**	-0.37**	-0.10
	K	0.18	0.45**	0.45**	0.85**	-0.19*	-0.38
2025	RH	-0.16	0.51**	0.80**	0.88**	0.29**	0.40**
	Ox	0.43	0.25	0.44**	-0.70**	0.38	-0.44**
	Acid	0.33**	0.79**	0.51**	0.41**	0.26**	0.57**
	K	0.21	0.34**	0.32**	0.73**	0.22**	0.48**

(*: $p<0.05$, **: $p<0.01$)

Table S5. Correlation analysis of SIA, Ws, RH and T during three periods in 2024.

	SIA		
2024	Pre-fireworks	Fireworks	Post-fireworks
Ws	-0.13	-0.52**	0.20**
RH	0.30	0.34**	0.42
T	0.37**	-0.31**	-0.38

(**: $p<0.01$)

Table S6. Statistical analysis of $PM_{2.5}$, SIA and CO in three years.

	CO		
	2023	2024	2025
$PM_{2.5}$	0.70**	0.84**	0.67**
SIA	0.44**	0.67**	0.60**

(**: $p<0.01$)

Table S7. Correlation analysis of $PM_{2.5}$, SIA and CO during three periods in 2023-2025.

		Pre-fireworks		Fireworks		Post-fireworks	
		$PM_{2.5}$	SIA	$PM_{2.5}$	SIA	$PM_{2.5}$	SIA
2023		0.85**	0.6*	0.68**	0.72**	0.67**	0.36*
2024	CO	0.88**	0.63**	0.79**	0.31**	0.75**	0.32**
2025		0.75**	0.68**	0.74**	0.62**	0.46**	0.22**

(*: $p<0.05$, **: $p<0.01$)

References

1. Chang, Y.; Kan, H.; Xie, M.; Deng, C.; Zou, Z.; Liu, S.; Zhang, Y. First long-term and near real-time measurement of trace elements in China's urban atmosphere: temporal variability, source apportionment and precipitation effect. Atmos. Chem. Phys. 2018, 18 (16), 11793–11812.
2. Norris, G.; Duvall, R.; Brown, S.; Bai, S.; EPA Positive Matrix Factorization (PMF) 5.0 Fundamentals and User Guide Prepared for the US Environmental Protection Agency Office of Research and Development Washington, DC. 2014.
3. Amato, F.; Hopke, P.K.; Source apportionment of the ambient $PM_{2.5}$ acrossst. Louis using constrained positive matrix factorisation. Atmos. Environ. 2012, 46, 329–337.
4. Hopke, P.K. Review of receptor modeling methods for source apportionment. J. Air Waste Manag. Assoc. 2016, 66, 237-259.
5. Li, H.; Wu, H.; Wang, Q.; Yang, M.; Li, F.; Sun, Y.; Qian, X.; Wang, J.; Wang, C. Chemical partitioning of fine particle-bound metals on haze-fog and non-haze-fog days in Nanjing, China and its contribution to human health risks. Atmos. Res. 2017, 183, 142-150.
6. Huang, R.; Cheng, R.; Jing, M.; Yang, L.; Li, Y.; Chen, Q.; Chen, Y.; Yan, J.; Lin, C.; Wu, Y.; Zhang, R.; El Haddad, I.; Prevot, A.S.H.; O'Dowd, C.O.; Cao, J. Source-specific health risk analysis on particulate trace elements: coal combustion and traffic emission as major contributors in wintertime Beijing. Environ. Sci. Technol. 2018, 52, 10967-10974
7. Lin, Y.; Zhang, Y.; Song, W.; Yang, X.; Fan, M. Specific sources of health risks caused by size-resolved PM-bound metals in a typical coal-burning city of northern China during the winter haze event. Sci. Total Environ. 2020, 734, 138651.
